# “An Even Playing Field”: Non-Clinical Simulation for Leadership Development Among Pediatric Interns

**DOI:** 10.12688/mep.21713.1

**Published:** 2026-08-06

**Authors:** Katherine D. Rosengard, Sabrina A. Karim, Thomas J. Sandora, Kelsey A. Miller

**Affiliations:** 1 Boston Children's Hospital, Boston, MA, USA

**Keywords:** Graduate Medical Education, Leadership, Simulation, Communication, Innovation

## Abstract

**Background:**

Graduate training programs are tasked with preparing trainees to lead clinical teams. Currently, there is little consensus on how to develop their interpersonal leadership skills, and pediatric programs tend to focus on senior residents. Targeting pediatric interns with strategies for developing interpersonal leadership skills provides more opportunity to consolidate and apply these skills during training.

**Objective:**

We aimed to understand how simulation, informed by cognitive load theory and psychological safety, facilitates the development of interpersonal leadership skills among pediatric interns.

**Methods:**

Pediatric interns participated in an outdoor wilderness simulation repeated 4 times between July–November 2022. The simulation and debrief focused on consensus decision-making and communication. To explore if and how simulation outside of clinical teams and devoid of clinical content fostered interpersonal leadership skills, we conducted semi-structured focus groups after each debrief. We conducted thematic analysis of the recorded debriefs and focus groups.

**Results:**

Forty nine of 53 eligible interns participated. Participants demonstrated learning about interpersonal leadership skills and began to build a leadership identity. Simulation facilitated this development through 3 core elements. Detaching the simulation from clinical content and settings *leveled the playing field* and *engendered experimentation and examination.* Despite this separation, the simulation prompted participants to *link to clinical spaces* through reflection on translating interpersonal leadership skills to clinical settings.

**Conclusion:**

Simulation outside of clinical teams and free of clinical content creates safe spaces for pediatric interns to develop interpersonal leadership skills. Earlier acquisition of these skills may facilitate leadership development over the course of pediatric training.

## Introduction

Leadership is increasingly recognized as a core physician competency requiring systematic development. ^1^ Major accreditation bodies require graduate medical education programs to teach leadership development, specifically focusing on interpersonal and communication skills required to lead clinical teams. Yet, agreement on the optimal educational approach is currently lacking. ^2^ ^,^ ^3^ Instead, residents often rely on unguided, informal, and ad hoc experiences to develop their interpersonal leadership skills, leaving some trainees unprepared to assume leadership roles when they graduate. ^4^

To address this gap, several residency programs have performed needs assessments and developed dedicated leadership curriculums, often for senior residents. ^5^ ^,^ ^6^ Training interns would facilitate earlier skill development and consolidation through experiential learning. ^7^ However, it is challenging to provide early-stage trainees with true leadership experience in the clinical setting given the need for progressive authority and responsibility over the course of residency training. In pediatrics in particular, trainees must navigate multiple layers of supervision designed to optimize patient safety, inherently limiting their leadership opportunities. Yet, patient- and family-centered care in pediatric medicine necessitates skills in consensus decision-making and effective team communication. ^8^ ^–^ ^10^

Simulation provides a safe environment for early-stage trainees to gain leadership experience without risking patient safety. Several needs assessments have specifically called for the use of simulation in leadership training. ^3^ ^,^ ^5^ Simulation has also been leveraged to develop leadership skills within established and ad hoc clinical teams. ^11^ ^–^ ^15^ Yet, the lack of theoretical frameworks and rigorous evaluation leaves educators with ongoing uncertainty around how to most effectively employ simulation as a tool for non-technical skill development. ^16^

We hypothesized that a simulation leveraging cognitive load theory and psychological safety could help early-stage trainees develop interpersonal skills. Cognitive load theory highlights how cognitive work extraneous to learning information about a concept can interfere with learning the information intrinsic to that concept. ^17^ Psychological safety refers to whether individuals view an environment to be conducive to interpersonally risky behaviors. ^18^ The aim of this study was to examine if, how, and what pediatric interns learn about interpersonal leadership skills from such a simulation.

## Methods

Using a post-positivist paradigm, we hypothesized that cognitive load theory and psychological safety could be used to design a simulation to develop interpersonal leadership skills in early-stage trainees. We examined individuals’ experiences using an inductive approach to thematic analysis.

### Simulation design

We developed a 2-hour session for pediatric interns to learn about leadership skills during a non-clinical simulation and facilitated debrief. We chose a non-clinical simulation to reduce extraneous cognitive load from the clinical context. ^18^ To promote psychological safety, we situated it during a twelve-week outpatient block during which participants develop personal familiarity with one another. We purchased Cascades Survival Simulation™, an exercise from Human Synergistics International, known for leadership development and team building. The wilderness survival simulation was chosen for its alignment with ACGME common program requirements for residents to achieve competency in *interpersonal and communication skills* and *teamwork*, key skills for leaders.

### Study setting

This study took place at Boston Children’s Hospital between July–November 2022. All pediatric interns attended one of four sessions, with 11–14 participants. During the 40-minute simulation, participants developed a group survival strategy for a wilderness disaster scenario. This was followed by a 40-minute facilitated debrief prompting participants to probe their consensus decision-making and interpersonal skills. The final 40 minutes were a semi-structured focus group (see Extended Data Supplement 1 for discussion guide) ^19^ exploring if and how non-clinical simulation fosters these skills. Participation in the simulation is part of the existing residency curriculum, so consent was not required. At the start of the focus groups, we obtained verbal consent rather than written consent to facilitate an efficient transition from the simulation into the debrief. We ensured participants were informed of the study purpose, voluntary nature of participation, confidentiality measures, and their right to decline or withdraw at any time. No participants opted out. The sessions were facilitated by one of two faculty co-directors (KAM, TJS) of a three-year longitudinal residency leadership curriculum. The Boston Children’s Hospital Institutional Review Board deemed the study exempt (IRB-P00027130).

### Data collection and analysis

The debriefs and focus group discussions were recorded, anonymized, and transcribed. Using an inductive approach, we conducted thematic analysis using Dedoose qualitative analytic software (Los Angeles, CA: SocioCultural Research Consultants, LLC), with the entire study team reviewing for coding consistency. We met regularly to consider relationships among codes to generate initial themes and to iteratively revise and refine both codes and themes. We engaged in dialogical reflexivity to challenge and question our own interpretations (see Extended Data Supplement 2 for detailed reflexivitiy reporting). ^19^ We continued coding and revising themes until we felt the themes could be stably named and defined with no new codes or themes emerging.

## Results

Forty-nine of 53 eligible interns participated. We identified that non-clinical simulation prompts early-stage trainees to experiment with and examine their actions and group interactions for leadership behaviors and opportunities, to link these behaviors and opportunities to clinical experiences, and to begin to build a leadership identity. We developed a visual representation of our themes ( Figure 1) highlighting the three core elements – leveling the playing field, engendering experimentation and examination, and linking to clinical spaces – that led to successful consensus decision-making and communication skill development. The two core elements of (1) leveling the playing field and (2) engendering experimentation and examination emphasize the broader theme that creating a safe space is key to interpersonal leadership skill development for early-stage trainees. We cite focus group quotations with the following annotation code: focus group number and participant number (example: participant 1–1 for speaker 1 in the first focus group). We use “X” when cross-talk precluded our identifying the speaker.

**Figure 1.**
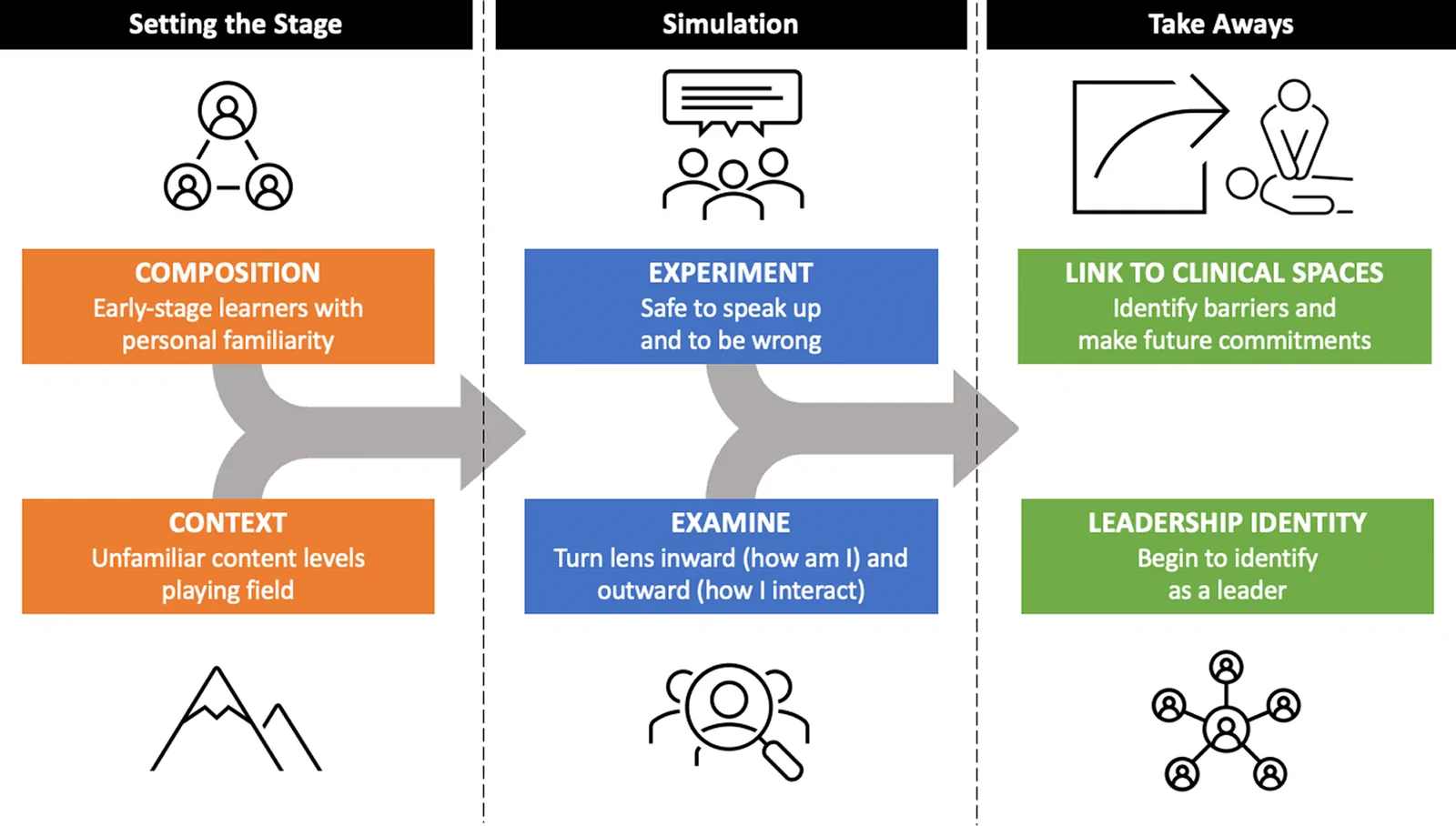
Non-Clinical Simulation Teaches Interpersonal Leadership to Early-Stage Trainees Through Experimentation and Guided Debriefing. This figure provides a visual representation of the key themes from our study and describes how those themes can be organized to outline the process through which an intentionally designed, non-clinical simulation can be leveraged to teach early-stage trainees about leadership skills and how to apply those skills in the clinical realm.

### Theme 1: Leveling the playing field

Participants emphasized the impact of both the composition and context of the simulation on their experiences and learning. They felt more comfortable that all members of the simulation were early in training and suggested that “if there were tiered experience levels … [it] would make [them] as the intern … less inclined to speak up” (Participant 2–1). They appreciated how their pre-established familiarity created mutual trust and respect. One participant dissected the benefits of personal familiarity, saying “this went well because we know each other, we trust each other, and we respect each other, and we felt comfortable sharing our thoughts, our lack of knowledge at times” (Participant 1–12). Their shared respect also gave participants the courage to “bring up points where [they] disagree[d] with the group … [which they] would have been a little bit less willing to do […] if [they] did not know anyone” (Participant 4–2).

Grounding the simulation in unfamiliar content outside of medicine led participants to feel they were all “on an even playing field [… because] it’s not about what [they] truly academically [knew]” (Participant 4–6). The non-clinical setting “lowered the stakes” (Participant 2–5) and obviated the perceived importance of pre-existing differences in knowledge or skills. There emerged a resulting willingness to soundboard freely with each other. Participants recognized unfiltered communication is “harder to do when you feel like you should know more” (Participant 1-X).

### Theme 2: engendering experimentation and examination

Participants described how this environment made it safe to experiment because although they were working with peers they knew from clinical spaces, they were not working in a clinical environment. They described feeling as though “there are right [answers in] medical situations” and so if someone is “wrong, it’s a poor reflection” (Participant 3–32) on that individual. Freed from fear of being wrong, the interns felt safe to say “I don’t know anything about this, but what do we think about this” (Participant 1-X). Thus, participants were able to experiment with new problem-solving strategies during the simulation.

Participants also felt the activity compelled them to examine their own behaviors and their interactions with others. Participants evaluated their contributions to the team and how those related to their personal decision-making and communication skills:

“I could be a bit more balanced […] I’m a big picture person” (Participant 2–6).

Exposure to the spectrum of decision-making and communication styles displayed across participants led individuals to consider how they could best leverage their own and others’ skills and expertise in working towards common goals. For example, participant 3–4 observed:

“You initially said ‘I also want to hear what everyone else thinks,’ [setting] the tone for everyone to be able to jump in.”

Taken together, themes 1 and 2 highlight how the non-clinical setting was a major driver of producing a safe space for leadership development. In addition to personal familiarity, the lack of clinical content and the separation from clinical environments were described as critical to residents’ comfort experimenting with and reflecting on interpersonal leadership skills early in medical training.

### Theme 3: linking to clinical spaces

The activity prompted participants to link their learnings within this safe space to clinical workplaces and experiences. Participants reflected on how they could and should apply lessons from this non-hierarchical, non-clinical simulation to clinical settings. This reflection involved contrasting their behaviors in simulation to their behaviors in clinical spaces and identifying potential barriers to replicating desired behaviors in clinical spaces. For example, participant 3–30 described how being removed from clinical spaces “enabled all of us to do a better job of seeking other people’s expertise and enabled people to move away from the assigned role, the ego, previous knowledge and biases that might be at play.” In contrast, roles in clinical settings can mask the fact that “within those tiers, people have levels of expertise” (Participant 2–7). Participants also recognized the need to break down the hierarchies innate to clinical work in order to facilitate consensus decision-making.

These reflections led participants to consider steps they could take to enact interpersonal leadership skills in clinical settings. Participants recognized how leading clinical teams with vulnerability could facilitate a safe space, similar to the environment they experienced in the simulation. Many participants made pledges about how they wanted to lead differently on clinical teams to create that sense of safety:

“When I’m starting on a new team, I’ll want to make sure that I’m vulnerable with them so that they know that they can be vulnerable to me” (Participant 1–13).

In turn, participants considered their leadership identity. As interns reflected on their participation in this exercise, they identified ways in which they all acted “inherently [as] leaders in some sense” and how they “shared the leadership responsibility” (Participant 3–8), despite leadership roles not being preassigned nor assigned by participants during the simulation. Instead, participants led organically when it aligned with their strengths. Reflecting on this led participants to newly identify themselves as potential clinical leaders, particularly on interprofessional teams.

## Discussion

Our findings illustrate the potential of non-clinical simulation to provide early-stage trainees an opportunity to reflect on and develop interpersonal leadership skills. Furthermore, participants recognized the transferability of skills learned in non-clinical simulation to clinical environments.

While the medical education community is still exploring how to best develop trainees’ leadership skills, simulation is repeatedly acknowledged as a helpful tool. ^11^ ^–^ ^15^ Previous efforts to develop leadership in pediatric trainees through simulation have utilized clinical simulations, often focusing on acute scenarios such as resuscitations or specific settings such as intensive care units. This study is novel in exploring how simulation separated from clinical medicine can be used for leadership development. While we initially chose a non-clinical simulation based on cognitive load theory to avoid distraction by medical content, ^18^ we learned that this decision was a key driver in cultivating a psychologically safe environment where participants could experiment without fear of being wrong and reflectively examine interpersonal skills (in themselves and their peers) that are critical to leadership.

Participants’ recognizing psychological safety as a catalyst for learning resonates with the literature. ^20^ Psychological safety creates environments where people can risk being wrong or ask for help – behaviors that are simultaneously interpersonally risky and critical to development. ^17^ For interpersonal leadership skill development, therefore, supportive and safe contexts seem fundamental. ^4^ ^,^ ^21^ Creating psychological safety may be particularly important for early-stage trainees, who report feeling intimidated by others, unsure of their roles, and focused primarily on “fitting in” as they transition from undergraduate to graduate medical education. ^22^ Our findings also generate new questions around how contributors to psychological safety might change throughout medical training. For novices, the separation from the clinical content and teams appears beneficial. For more experienced trainees, further study should assess the value of practicing interpersonal leadership skills in non-clinical environments.

Much has been written about the need for healthcare leaders to foster psychological safety in clinical environments. ^23^ It would be ideal, therefore, if experiencing psychological safety as they develop interpersonal leadership skills could help physicians learn to foster psychological safety once they become clinical leaders. And that is, indeed, what we saw in our study. In reflecting on how to transfer their learning from non-clinical settings, participants focused primarily on how to foster the safe space they experienced in this non-clinical simulation. They also grappled with identified barriers to psychological safety in healthcare settings, such as hierarchy and professional role boundaries. ^23^ ^,^ ^24^ Although we did not formally assess transferability, studies of more experienced physicians and of non-medical professionals show that leadership skills learned in psychologically safe non-work environments are transferrable to more stressful, hierarchical authentic workspaces. ^25^ ^,^ ^26^ Future study should confirm if and to what extent this finding is also true for early-stage learners such as pediatric interns.

Our study has limitations. Taking place within a single, large pediatric residency program may limit generalizability. Leadership curriculum directors facilitated the simulations, potentially affecting what participants said in the debriefs and focus groups despite the lack of any evaluative component to the activity. However, these faculty members brought consistency with deep knowledge of the exercise and leadership development, and prior experience with conducting simulations for trainees. While we intentionally adopted an inductive coding approach, it is possible that the theoretical underpinnings of the simulation influenced the perspectives of the two coders who were aware of the theoretical basis. We strove to protect against this by including two coders who had no knowledge of the theoretical concepts informing the simulation design and by regular discussions among all team members in which we questioned and delved deeper into each other’s interpretations of the data.

## Conclusion

Our study contributes to a broader understanding of how simulation can be used for intentional leadership education, identifying non-clinical simulation as a beneficial tool for helping develop interpersonal leadership skills including consensus decision-making and communication early in training. The non-clinical context and early-stage trainee composition of the simulation provided a safe space for experimenting with and examining actions and group interactions for leadership behaviors. Despite being separate from clinical content and environment, trainees demonstrated the ability to link new interpersonal leadership skills and a nascent leadership identity to clinical settings. This study can equip educators to advance leadership development for physicians-in-training in a more theoretically informed way during early stages of training.

## Data accessibility

The data generated and analyzed during this study are not publically available. The study was deemed exempt by the Boston Children’s Hospital Institutional Review Board (12/15/2022, IRB-P00027130), and the authors assured the IRB in that application that the data would not be reused or disclosed. Further, the authors are bound by copyright protections and cannot reveal details of the Cascades Survival Simulation™, owned by Human Synergistics International. However, the authors’ facilitator guide and reflexivity exercise data is available (see manuscript references):

19. Rosengard KD, Karim SA, Sandora TJ, Miller KA. Extended Data for “An Even Playing Field”: Non-Clinical Simulation for Leadership Development Among Pediatric Interns. *Zenodo.* Preprint posted online July 10, 2026. doi: 10.5281/ZENODO.21299303
