# Supplementary material for: “An Even Playing Field”: Non-Clinical Simulation for Leadership Development Among Pediatric Interns
Source: MedEdPublish (2016). 2026 Oct 10;16:37. Originally published 2026 Aug 6. [Version 2] doi: 10.12688/mep.21713.2 (PMC13691357; doi:10.12688/mep.21713.2)
Supplement: Peer review history

# Reviewer report: Reviewer response for version 2

**DOI:** 10.21956/mep.23480.r46966

**Published:** 2026-10-22

**Authors:** Bibi Sumera Keenoo

**Affiliations:** 1 University of Mauritius, Réduit, Moka, Mauritius

This brief report explores how a non-clinical wilderness survival simulation may facilitate interpersonal leadership learning among paediatric interns.  The authors describe a two-hour educational intervention comprising a simulated group decision-making exercise, facilitated debriefing, and a subsequent focus-group discussion.  The authors analysed qualitative data from 49 interns across four sessions, thematically identifying three interrelated themes: levelling the playing field, engendering experimentation and examination, and linking learning to clinical spaces.

 The study addresses an important issue in postgraduate medical education: how to introduce leadership development early in residency training, particularly when hierarchical clinical environments may limit junior trainees’ opportunities to exercise leadership.

 The manuscript is clearly written, educationally relevant and supported by an appropriate exploratory qualitative approach.  The use of cognitive load theory and psychological safety provides a useful rationale for the educational intervention.  The participant quotations offer valuable insights into how interns experienced the activity and reflected on their interpersonal interactions.

 I also appreciate the authors' constructive responses to previous reviewers and the improvements incorporated into Version 2, particularly the clarification of the study objective and the acknowledgement that the study did not formally assess transfer of learning to clinical settings

 Nevertheless, several issues warrant further clarification to strengthen methodological transparency and ensure that the conclusions remain proportionate to the evidence presented.

 Major comments

**1.  Distinguishing perceived learning from demonstrated leadership skill development**

 The central issue is distinguishing participants’ perceptions of learning from evidence of actual leadership skill acquisition.

 The qualitative findings demonstrate that participants perceived the non-clinical simulation as providing opportunities to communicate, experiment with group decision-making, reflect on interpersonal behaviours and consider their emerging leadership identities.

 However, the study did not directly assess changes in leadership competence, observable behaviours or subsequent performance in clinical environments.  The study included no pre- and post-intervention assessments, independent behavioural observations, or longitudinal evaluations.

 Consequently, interpret statements suggesting that the intervention demonstrated leadership skill development cautiously.

 I recommend that the authors consistently distinguish between perceived learning, reflection, and intended behavioural change on the one hand, and demonstrated leadership competence on the other.

 In particular, the abstract, Figure 1, and conclusion should be reviewed to ensure that claims about developing interpersonal leadership skills do not exceed what the qualitative data reasonably support.

 This clarification is important for scientific validity and should be addressed.

**2   Greater transparency regarding the thematic analysis**

 The manuscript reports an inductive thematic analysis informed by a post-positivist paradigm, with coding consistency checks and dialogical reflexivity.

 While these elements suggest a considered analytical process, further methodological clarification would strengthen the credibility and reproducibility of the findings.

 Specifically, the authors should clarify:

- Which particular approach to thematic analysis was adopted and how it aligned with the stated research paradigm.
- Whether coding was conducted independently or collaboratively, and how the authors addressed differences in interpretation.
- How the initial codes were developed into the final themes.
- How the authors managed the theoretical assumptions informing the intervention during the inductive analysis.
- Whether the debriefing data and focus-group data were analysed separately at first or combined throughout the analysis.

The authors also indicate that analysis continued until no new codes or themes emerged.   It would help to clarify how they reached this decision and why this criterion fit their chosen analytical approach.

 These clarifications would improve methodological rigour without necessarily requiring additional data collection or analysis.

**3.  Influence of facilitator roles and social desirability**

 Faculty members who were also co-directors of the residency leadership curriculum facilitated the simulation sessions and discussions.

 The authors appropriately acknowledge that this relationship may have influenced participants’ responses.  However, the authors could explore the implications for data generation and interpretation more explicitly.

 Interns may have been inclined to express favourable views of the intervention or describe leadership-related learning in ways they perceived to be educationally desirable, particularly when discussing their experiences with faculty involved in their training.

 I recommend that the authors clarify whether participation in the research discussion was clearly separated from routine educational evaluation, and explain any measures taken to minimise perceived pressure or socially desirable responses.

 The authors should also consider the potential influence of the facilitators on the discussions and resulting themes when interpreting the findings.

**4.  Interpretation of psychological safety**

 Psychological safety is central to themanuscript’ss conceptual framework and interpretation of the results.

 Participants’ accounts provide persuasive evidence that they found the simulation relatively comfortable and conducive to open discussion. However, the study did not independently measure psychological safety, and the design does not establish that removing clinical content alone produced this experience.

 Other intervention features may have contributed, including participants’ familiarity, similar training levels, and the absence of formal assessment and facilitation.

 I recommend that the authors more explicitly distinguish between perceived psychological safety and objectively demonstrated effects attributable to specific intervention components.

 Rather than implying that non-clinical content independently created psychological safety, the findings could suggest that several interacting design characteristics contributed to an environment participants perceived as supportive of experimentation and reflection.

 This more nuanced interpretation would strengthen the manuscript’s conceptual contribution.

**5.  Clarification of the relationship between reflection and clinical transfer**

 The third theme, linking to clinical spaces, is particularly relevant to thestudy’s educational implications.

 Participants demonstrated an ability to identify potential applications of their experiences to clinical teamwork.  However, identifying possible applications and expressing intentions to behave differently should not be equated with successful transfer of learning to clinical practice.

 The revised discussion appropriately acknowledges that transferability was not formally assessed.

 I suggest maintaining this distinction consistently throughout the manuscript, particularly in the abstract and conclusion.

 The findings support the proposition that non-clinical simulation may stimulate reflection on future clinical leadership behaviours.  Whether these reflections translate into sustained behavioural changes remains an important question for subsequent research.

 Minor comments and suggestions

**1   Description of the educational intervention**

 The manuscript provides the intervention’s broad structure and duration, and the supplementary facilitator materials are useful.

 However, it would help to provide a concise description of the specific interpersonal leadership behaviours the exercise targeted and how the debrief addressed them.

 Given the simulation’s proprietary nature, a clearer description of the general educational principles and debriefing strategies would help educators adapt the approach without reproducing copyrighted material.

**2.  Leadership identity formation**

 The manuscript suggests that participants began to recognise themselves as potential leaders.

 This is an interesting finding, although the evidence primarily reflects immediate self-reported recognition and reflection.

 The authors could clarify that the study provides preliminary insights into emerging leadership identity rather than evidence of sustained professional identity formation.

**3   Limitations and transferability**

 The limitations section appropriately acknowledges the single-institution setting, possible facilitator influence and the theoretical perspectives of the researchers.

 It would also be useful to acknowledge the reliance on immediate post-intervention reflections, the lack of longer-term follow-up, and the absence of direct assessment of leadership behaviours.

 These limitations are particularly relevant when considering the potential application of the findings to other specialities, stages of training and clinical environments.

**4.  Data availability and reproducibility**

 The authors explain that they cannot make the underlying qualitative data publicly available because of commitments made during institutional ethical review.  The availability of supplementary facilitator and reflexivity materials is appreciated.

 However, because the complete transcripts are unavailable, independent verification of the coding and thematic interpretations is necessarily limited.

 I recommend that the authors ensure the data availability statement clearly distinguishes between the underlying empirical data that cannot be shared and the publicly accessible supplementary methodological material.

 The ethical restrictions should be respected, and the authors are not requesting disclosure of identifiable participant data.

**5.  Alignment between Figure 1 and the findings**

 Figure 1 provides a useful visual synthesis of the proposed educational process.

 However, the figure may be interpreted as depicting an established causal pathway from theintervention’ss design features to leadership skill development.

 I suggest that the authors present it explicitly as an interpretive model derived from participants’ reported experiences, rather than a validated model of causal mechanisms or demonstrated learning outcomes.

 Conclusion and recommendation

 Overall, this is a worthwhile and educationally relevant study addressing an important aspect of leadership development among early-stage medical trainees.  The qualitative findings offerfindings offer useful insights into how non-clinical simulation may create opportunities for interpersonal experimentation, nreflection and consideration of future leadership behaviours.

 The study design suits its exploratory aims, and the manuscript contributes usefully to discussions of simulation-based leadership education.

 My principal reservations concern the distinction between perceived learning and demonstrated competence, the transparency of the qualitative analytical process, and the interpretation of psychological safety and transferability.

 The first two issues, along with ensuring that the abstract, conceptual figure, and conclusions align with the evidence, are particularly important for scientific soundness.  The remaining points are intended to improve methodological clarity and interpretive precision.

 I believe these concerns can reasonably be addressed through targeted revisions and clarifications, without requiring a fundamentally different study design.

Have any limitations of the research been acknowledged?

Yes

Is the study design appropriate and does the work have academic merit?

Yes

Is the work clearly and accurately presented and does it cite the current literature?

Yes

If applicable, is the statistical analysis and its interpretation appropriate?

Not applicable

Are all the source data underlying the results available to ensure full reproducibility?

Partly

Are the conclusions drawn adequately supported by the results?

Partly

Are sufficient details of methods and analysis provided to allow replication by others?

Partly

Reviewer Expertise:

Health Professions Education; Medical Education; Healthcare Professionalism; Clinical Leadership and Professional Development; Qualitative Research; Teaching and Learning in Health Professions.

I confirm that I have read this submission and believe that I have an appropriate level of expertise to confirm that it is of an acceptable scientific standard, however I have significant reservations, as outlined above.

## References

[1] : Using thematic analysis in psychology. *Qualitative Research in Psychology* .2006;3(2) : 10.1191/1478088706qp063oa77-101 10.1191/1478088706qp063oa

[2] : Conceptual and design thinking for thematic analysis. *Qualitative Psychology* .2022;9(1) : 10.1037/qup00001963-26 10.1037/qup0000196

# Reviewer report: Reviewer response for version 2

**DOI:** 10.21956/mep.23480.r46942

**Published:** 2026-10-12

**Authors:** Heather L. Newton

**Affiliations:** 1 Eastern Virginia Medical School, Norfolk, Virginia, USA

The implemented revisions were well done, as suggested by the other reviewer.

Have any limitations of the research been acknowledged?

Yes

Is the study design appropriate and does the work have academic merit?

Yes

Is the work clearly and accurately presented and does it cite the current literature?

Yes

If applicable, is the statistical analysis and its interpretation appropriate?

Yes

Are all the source data underlying the results available to ensure full reproducibility?

Partly

Are the conclusions drawn adequately supported by the results?

Yes

Are sufficient details of methods and analysis provided to allow replication by others?

Yes

Reviewer Expertise:

Graduate Medical Education and pediatric residency programs. I am an EdD, MEd, and MBA educator for our institution. I have done research on clinical leadership, specifically with pediatric residents - with an ongoing 10 year project in this field.

I confirm that I have read this submission and believe that I have an appropriate level of expertise to confirm that it is of an acceptable scientific standard.

# Reviewer report: Reviewer response for version 1

**DOI:** 10.21956/mep.23261.r46398

**Published:** 2026-08-19

**Authors:** Jan Frich

**Affiliations:** 1 Faculty of Medicine, University of Oslo, Oslo, Norway

The literature on leadership development in healthcare is growing, and systematic reviews suggests that experiential learning and project work is associated with positive outcomes (Lyons et al. 2021). Simulation is being used in leadership development, but the literature is limited. Also, simulation for medical doctors outside the clinical setting is novel approach. The study is explorative, using qualitative methods. 

 This manuscript spells out two aims: "to understand how simulation, informed by cognitive load theory and psychological safety, facilitates the development of interpersonal leadership skills among pediatric interns." (abstract). In the introduction: "The aim of this study was to examine if, how, and what pediatric interns learn about interpersonal leadership skills from such a simulation." 

 Some suggestions for improvement: 

 I think it is better if the aim is spelled out in the same way to be clear and focused. I suggest picking one of these. I would suggest: "to understand how simulation outside a clinical setting may facilitate interpersonall leadership skills among pediatric interns". Furhter; I will suggest that the approach and content is described not in the aim. 

 Psychological safety. I think it may be better to write that the simulation program is informed by the prinicples of psychological safety. 

 Methods: "Using a post-positivist paradigm, we hypothesized that cognitive load theory and psychological safety could be used to design a simulation to develop interpersonal leadership skills in early-stage trainees." I suggest deleting the first part "Using a post-positivist paradigam". Start with: "We found a qualitative approach appropriate and hypothesized that cogntive load theory combined with the principles of psychological safety ...."

 The study setting and methods are described in sufficient detail, and the methods for data collection are approproiate. It is a token of quality that several researchers have analyzed the data. 

 The results focus much on the setting and the climate (psychological safety), and less on interpersonall skills. Is there any more data that could elict participatns' non-technical learning outcomes and identity formation? (see a discussion of identity processes in Spehar et al 2015)

 Discussion:  The authors could be more specific on the particular outcomes of doing leadership development in this setting. Could the same feeling of psychological safety be promoted in clinical settings?  

 I am not able to download Figure 1, so I cannot assess this.

 Under data accessability the full reference could be deleted. A reference to the number in the reference list may be sufficient. 

Have any limitations of the research been acknowledged?

No

Is the study design appropriate and does the work have academic merit?

Yes

Is the work clearly and accurately presented and does it cite the current literature?

Yes

If applicable, is the statistical analysis and its interpretation appropriate?

Not applicable

Are all the source data underlying the results available to ensure full reproducibility?

No

Are the conclusions drawn adequately supported by the results?

Yes

Are sufficient details of methods and analysis provided to allow replication by others?

Yes

Reviewer Expertise:

Leadership in health care; health services research

I confirm that I have read this submission and believe that I have an appropriate level of expertise to confirm that it is of an acceptable scientific standard, however I have significant reservations, as outlined above.

## References

[1] : Evidence-based medical leadership development: a systematic review. *BMJ Leader* .2021;5(3) : 10.1136/leader-2020-000360206-213 10.1136/leader-2020-000360 37850339

[2] : Professional identity and role transitions in clinical managers. *Journal of Health Organization and Management* .2015;29(3) : 10.1108/JHOM-03-2013-0047353-366 10.1108/JHOM-03-2013-0047 25970529

# Response

**Published:** 2026-10-02

**Authors:** Katherine Rosengard

**Affiliations:** Not Applicable, USA

Thank you for your thoughtful review of our paper. You clearly took the time to understand the current literature on our topic, carefully review our findings and suggest additional areas worth investigating. We appreciate your time. We will provide point-by-point comments based on your suggestions below.   Your comments for our abstract & introduction:

- "This manuscript spells out two aims: "to understand how simulation, informed by cognitive load theory and psychological safety, facilitates the development of interpersonal leadership skills among pediatric interns." (abstract). In the introduction: "The aim of this study was to examine if, how, and what pediatric interns learn about interpersonal leadership skills from such a simulation." 
- I think it is better if the aim is spelled out in the same way to be clear and focused. I suggest picking one of these. I would suggest: "to understand how simulation outside a clinical setting may facilitate interpersonall leadership skills among pediatric interns". Furhter; I will suggest that the approach and content is described not in the aim. 
- Psychological safety. I think it may be better to write that the simulation program is informed by the prinicples of psychological safety.

Our response:

- Thank you for highlighting potential confusion in our differing descriptions of the aims. We've edited the abstract and introduction to clarify and focus our aims. We agreed that it was cleanest to remove specific mentions of cognitive load theory and psychological safety from the specific aims/hypothesis statements. 

Your comments on our methods:

- Using a post-positivist paradigm, we hypothesized that cognitive load theory and psychological safety could be used to design a simulation to develop interpersonal leadership skills in early-stage trainees." I suggest deleting the first part "Using a post-positivist paradigam". Start with: "We found a qualitative approach appropriate and hypothesized that cogntive load theory combined with the principles of psychological safety ...."
- The study setting and methods are described in sufficient detail, and the methods for data collection are approproiate. It is a token of quality that several researchers have analyzed the data.

Our response:

- Thank you for the recommendation. We feel that it is important to be specific about our paradigm as hypothesis-driven research is not aligned  with all qualitative methods. Furthermore, given the variety of different qualitative methods (e.g. grounded theory, ethnography, content analysis, reflexive thematic analysis, phenomenology, etc.), it feels inadequate to refer to  qualitative methods as a single approach. 
- Thank you, we are glad you appreciated the work we did to ensure our methods were sound.

Your comments on our results:

- The results focus much on the setting and the climate (psychological safety), and less on interpersonall skills. Is there any more data that could elict participatns' non-technical learning outcomes and identity formation? (see a discussion of identity processes in Spehar et al 2015)
- We agree with the reviewer's observation that assuming leadership roles can require identity work. This is an important area of future research, but was not a focus of this current study. We have added this for consideration as an important area of future study in the discussion section.

Your comments on our discussion:

- The authors could be more specific on the particular outcomes of doing leadership development in this setting. Could the same feeling of psychological safety be promoted in clinical settings?

Our response:

- We agree that this would be an important area of future research. We have edited the second to last paragraph of our discussion to further highlight that our study was not designed to assess transferrability of leadership skills or skills for creating psychologically safe environments. We hypothesize that they are but recommend future studies assess if and how transferrable these skills are.

Global comments:

- I am not able to download Figure 1, so I cannot assess this.
- Under data accessability the full reference could be deleted. A reference to the number in the reference list may be sufficient

Our response:

- Thank you for identifying an technical error that occurred during publishing. The MedEdPublish team has resolved it, and the figure is now available.
- We have reviewed the data accessibility statement to remove the full reference.

Thank you again for your time and careful consideration of our manuscript.

# Reviewer report: Reviewer response for version 1

**DOI:** 10.21956/mep.23261.r46403

**Published:** 2026-08-19

**Authors:** Heather L. Newton

**Affiliations:** 1 Eastern Virginia Medical School, Norfolk, Virginia, USA

This was a very well-written article and could easily be replicated using the Cascades Survival Simulation. Although the authors were not able to provide the data due to IRB constraints, they did provide the information for the extended data. They linked the article for the facilitator questions, which was helpful to read. I appreciated how they referred to the alignment of the ACGME common program requirements to achieve competency in interpersonal and communication skills and teamwork. If the residents are going to dive deep into this type of simulation, it makes sense to use material that will allow them forward trajectory to completing their residency milestones. 

Have any limitations of the research been acknowledged?

Yes

Is the study design appropriate and does the work have academic merit?

Yes

Is the work clearly and accurately presented and does it cite the current literature?

Yes

If applicable, is the statistical analysis and its interpretation appropriate?

Yes

Are all the source data underlying the results available to ensure full reproducibility?

Partly

Are the conclusions drawn adequately supported by the results?

Yes

Are sufficient details of methods and analysis provided to allow replication by others?

Yes

Reviewer Expertise:

Graduate Medical Education and pediatric residency programs. I am an EdD, MEd, and MBA educator for our institution. I have done research on clinical leadership, specifically with pediatric residents - with an ongoing 10 year project in this field.

I confirm that I have read this submission and believe that I have an appropriate level of expertise to confirm that it is of an acceptable scientific standard.

# Response

**Published:** 2026-10-02

**Authors:** Katherine Rosengard

**Affiliations:** Not Applicable, USA

Thank you for your time and effort in reviewing our manuscript. We appreciate your appreciation for our work and we are glad you found it practically relevant to trainees.
